# Transcriptomic response of the Antarctic pteropod *Limacina helicina antarctica* to ocean acidification

**DOI:** 10.1186/s12864-017-4161-0

**Published:** 2017-10-23

**Authors:** Kevin M. Johnson, Gretchen E. Hofmann

**Affiliations:** 1 Department of Ecology, Evolution and Marine Biology, University of California, Santa Barbara, Santa Barbara, CA 93106-9620 USA; 2 Department of Biological Sciences, Louisiana State University, Baton Rouge, LA 70803-0001 USA

**Keywords:** *Limacina helicina antarctica*, Pteropod, Ocean acidification, Ross Sea, pH, Southern Ocean, RNAseq, Gene expression

## Abstract

**Background:**

Ocean acidification (OA), a change in ocean chemistry due to the absorption of atmospheric CO_2_ into surface oceans, challenges biogenic calcification in many marine organisms. Ocean acidification is expected to rapidly progress in polar seas, with regions of the Southern Ocean expected to experience severe OA within decades. Biologically, the consequences of OA challenge calcification processes and impose an energetic cost.

**Results:**

In order to better characterize the response of a polar calcifier to conditions of OA, we assessed differential gene expression in the Antarctic pteropod, *Limacina helicina antarctica*. Experimental levels of *p*CO_2_ were chosen to create both contemporary pH conditions, and to mimic future pH expected in OA scenarios. Significant changes in the transcriptome were observed when juvenile *L. h. antarctica* were acclimated for 21 days to low-pH (7.71), mid-pH (7.9) or high-pH (8.13) conditions. Differential gene expression analysis of individuals maintained in the low-pH treatment identified down-regulation of genes involved in cytoskeletal structure, lipid transport, and metabolism. High pH exposure led to increased expression and enrichment for genes involved in shell formation, calcium ion binding, and DNA binding. Significant differential gene expression was observed in four major cellular and physiological processes: shell formation, the cellular stress response, metabolism, and neural function. Across these functional groups, exposure to conditions that mimic ocean acidification led to rapid suppression of gene expression.

**Conclusions:**

Results of this study demonstrated that the transcriptome of the juvenile pteropod, *L. h. antarctica*, was dynamic and changed in response to different levels of *p*CO_2_. In a global change context, exposure of *L. h. antarctica* to the low pH, high *p*CO_2_ OA conditions resulted in a suppression of transcripts for genes involved in key physiological processes: calcification, metabolism, and the cellular stress response. The transcriptomic response at both acute and longer-term acclimation time frames indicated that contemporary *L. h. antarctica* may not have the physiological plasticity necessary for adaptation to OA conditions expected in future decades. Lastly, the differential gene expression results further support the role of shelled pteropods such as *L. h. antarctica* as sentinel organisms for the impacts of ocean acidification.

**Electronic supplementary material:**

The online version of this article (10.1186/s12864-017-4161-0) contains supplementary material, which is available to authorized users.

## Background

Comparative transcriptomics has proven to be a powerful tool for examining organism-environment interactions in non-model species [1]. This approach has been applied in both laboratory and field settings, and has become a common method with which to assess physiological responses in marine taxa [2–5]. The advantages of comparative transcriptomics are particularly valuable in marine systems, where differential gene expression analysis has been used to explore how organisms respond to their abiotic environment. Examples include studies on adaptations to thermal stress [2, 3], population-level variation in tolerance [4, 5], and increasingly, studies conducted in a global change context [1, 6, 7]. In this study, we use the analysis of differential gene expression to examine the resistance of a key member of the zooplankton in the Southern Ocean, the shelled pteropod *Limacina helicina antarctica*, to the impacts of an advancing anthropogenic stress, ocean acidification.

Polar seas, such as the Southern Ocean, are predicted to be ‘first in time’ to experience the impacts of ocean acidification, the reduction of pH and saturation states for minerals such as aragonite as a result of the absorption of CO_2_ into surface waters [8–10]. Future acidification will be layered onto patterns of natural, present-day pH variability, with the cold waters of polar seas tending to hold and absorb more CO_2_ [11]. Thus, in polar waters, future anthropogenic ocean acidification will change carbonate chemistry sooner in time than in temperate waters. In the Southern Ocean, recent studies into variability in present-day pH in nearshore Antarctic regions have observed seasonal variability in pH dynamics [12]. Here, in winter, low-pH conditions (due to high CO_2_ levels) create seawater that is under-saturated with respect to aragonite, the form of calcium carbonate that makes up shells *L. h. antarctica.* In summer, a significant alkalization event has been observed [12], likely the result of photosynthesis and CO_2_ draw-down driven by summer phytoplankton blooms. In addition to this seasonal variability, regions of the Southern Ocean are predicted to experience ocean acidification, and hence aragonite under-saturation conditions in the winter as early as the year 2018 [8, 9, 12]. As ocean acidification progresses, models predict rapid changes in the next 10–20 years for many regions of the Southern Ocean with significant aragonite undersaturation on an annual basis for as much as 6 months of the year by the year 2050 [9, 12]. Overall, this rapid rate of change for the Southern Ocean will present a physiological and energetic challenge to calcifying marine organisms such as the shelled pteropod*s*.

Our study organism, the pteropod *Limacina helicina antarctica* (Phipps, 1774), is an ideal organism to assess organismal response to future ocean acidification in Antarctic waters. *L. h. antarctica* is a holoplanktonic mollusk, and a dominant member of the macrozooplankton assemblages in the Southern Ocean [13–15]. The thin calcium carbonate shells of *Limacina*, and pteropods in general, are particularly vulnerable to dissolution in the under-saturated conditions that are created in low pH seawater [16, 17]. Thus, pteropods are proposed as sentinel species for the impacts of ocean acidification [18] due to their aragonitic shell, a comparatively soluble for of calcium carbonate, which renders them more susceptible to dissolution in nature in under-saturated seawater [10, 13, 19]. Notably, recent assessments using scanning electron microscopy (SEM) of shell condition of *Limacina spp.* from both the Eastern Pacific and Southern Ocean shows that contemporary patterns of undersaturation are already causing shell dissolution [13, 14, 16]. With regard to *L. h. antarctica*, several investigators have noted that dissolution of the shell surface was detected in the majority of recently collected samples [14, 16]. Although researchers have made shipboard collections and analyzed preserved shells of *Limacina* from the Antarctic, few studies have examined the physiological consequences of maintaining net calcification in undersaturated conditions [20, 21].

Comparative transcriptomics is an ideal approach to examine the physiological underpinnings of the response of pteropods to dissolution stress [22, 23]. In this study, we present a comparison of transcriptomes from *L. h. antarctica* that were maintained in a short-term experiment at three environmentally-relevant pH treatments: (i) present-day summer (pH 8.13), (ii) present-day winter (pH 8.01), and (iii) future winter (pH 7.71) [12]. These experimental conditions allowed us to examine the seasonal effects of annual alkalization events, and the predicted effects of ocean acidification conditions on the transcriptome of *L. h. antarctica*. Analysis of the data revealed that *L. h. antarctica* responds to *p*CO_2_ changes with rapid alterations of the transcriptome that impacted the expression of genes involved in several relevant physiological processes.

## Methods

### Field collection of animals

Juvenile pteropods (*Limacina helicina antarctica*) were collected through the sea ice in McMurdo Sound in the southern Ross Sea on October 12^th^, 2015. Pteropod collections were performed at a depth of 50 m using a fixed-frame bongo net (50 cm × 150 cm net with 333 μm mesh; cod ends with 200 μm and 333 μm mesh) deployed through a dive hole drilled in the sea ice at a near-shore site on the sea ice (77°50′53″ S, 166°35′59″ E). The bongo net was equipped with 2 small floats attached at the cod end to prevent the ends from pinching in low current. In addition, a swiveling double shackle was used to attach the rotating towing yoke on the bongo net to the end of the winch line. This allowed the net to rotate into a horizontal position in the water column and maintain proper orientation with the currents during the deployment period. Bongo depth was estimated using a meter wheel to deploy 50 m of line.

### Post-collection handling

After collection, pteropods from a single tow were maintained in ambient seawater (−1.2 °C) in a 19-l collection container, and quickly transported to the Crary laboratory facilities at McMurdo Station, the U.S. research station on Ross Island. Individuals used in the experimental run were maintained in seawater tables at −1.2 °C for 48 h prior to being added to the pre-equilibrated CO_2_ exposure zooplankton culturing system.

### CO_2_ exposure experiment

For the CO_2_ exposure experiment, pteropods were held in a flow-through culturing system designed to have controlled levels of *p*CO_2_ with sufficient flow to maintain macrozooplankton in long-term experiments. The system is based upon the generation of reservoir seawater sources that are connected to replicate culture tanks for each treatment. The reservoir mixing system that generated the target experimental *p*CO_2_ levels was fabricated following previously published methods [24]. Briefly, filtered, CO_2_-scrubbed, dry air was mixed with pure CO_2_ using a SmartTrak® 100 Series Mass Flow Controller and a MicroTrak™ 101 Series Mass Flow Controller (Sierra Instruments, USA), respectively. These reservoirs were held in a 1240-l sea table that kept the treated seawater at the desired experimental temperature (−1.2 °C). The *p*CO_2_ treated seawater was then pumped into the zooplankton flow-through culturing system.

The zooplankton culture system, henceforth referred to as the Z-system, consisted of 18 culture vessels that were submerged in ambient seawater surrounding the reservoir tanks. Each zooplankton culture vessel was a clear 1-l polycarbonate plastic tank and lid (AHLT3, Aquatic Ecosystems, USA) originally designed for rearing juvenile zebrafish. Each tank contained a pair of 400 μm mesh baffles (AHLB400, Aquatic Ecosystems, USA) to prevent pteropods from escaping. To ensure that the animals were exposed to a continuous flow of *p*CO_2_ treated seawater, water input was regulated at a flow rate of 2.0 l per hour. The volume of the 1-l tanks was replaced every 30 min. As a result, the *p*CO_2_ levels in the culture vessels were highly similar within a treatment, and closely tracked the reservoir *p*CO_2_ concentrations.

The experimental *p*CO_2_ treatments were designed to create two conditions: (1) pH ranges that bracketed contemporary annual pH variability, and (2) pH conditions expected to occur under scenarios of ocean acidification for the Southern Ocean. Here, treatment conditions for contemporary pH were based on observations of oceanic pH in McMurdo Sound during the 2013–2014 summer [12]. These selected pH values, representing a range of current and predicted ranges of *p*CO_2_ levels, pH, and aragonite saturation states are summarized in Table 1. Pteropods were not fed during the course of this experiment as the winter-to-summer transition in the McMurdo Sound is characterized by limited phytoplankton abundance [25].

**Table 1:** pCO_2_ treatment conditions

Scenario	Temperature (°C)	pCO_2_ (μatm)	pH	Ω aragonite
Present-Day Summer	−0.98 ± .06	318 ± 46	8.13 ± .05	1.67 ± .14
Present-Day Winter/ Future Summer	−0.98 ± .07	432 ± 44	8.01 ± .03	1.30 ± .09
Future Winter	−0.98 ± .07	902 ± 49	7.71 ± .02	0.68 ± .03

### Carbonate chemistry

Seawater chemistry for the Z-system was measured for each experimental tank on a daily basis. Temperature was measured using a wire thermocouple (HH81A, Omega), and salinity was measured using a conductivity meter (YSI 3100, USA). Z-system pH was calculated for each tank from a 20 ml sample measured using a spectrophotometer (Bio Spec-1601; Shimadzu) and the indicator dye m-cresol following the standard operating procedure (SOP) 6b [26]. Seawater samples (in 500 ml borosilicate bottles) were collected directly from the reservoir vessels every 3 days; in preparation for measuring total alkalinity (T_A_), the bottle sample was immediately poisoned with 0.02% mercuric chloride, and stored at +4 °C until analyzed. T_A_ from these preserved samples was measured using an open-cell titration method with a Mettler-Toledo T50 titrator (Mettler Toledo, USA) using a 0.1 mol l^−1^ HCl in seawater titrant (Dickson Laboratory, Scripps Institute, USA). Measurements of T_A_ were verified with a certified reference material (CRM) seawater standard [27]. From these measurements carbonate chemistry parameters were calculated using the software CO_2_calc [28].

### Sample collection and processing

During the experiment, pteropods were collected at the 5 time points: Time zero (T_0_, collected immediately preceding the addition of animals to the Z-system), and then at 4 intervals after the start of the experiment on days 1, 7, 14, and 21. In addition to these sampling time-points, mortality checks were conducted daily.

### RNA extraction and sequencing

When collecting from the animals from the Z-system tanks, 10 actively swimming pteropods were quickly placed into a single 1.5 ml micro-centrifuge cryovial, excess seawater was removed, and the sample was then flash frozen in liquid nitrogen. Sample vials were subsequently stored at −80 °C until RNA extraction. Due to the small size of juvenile *L. h. antarctica*, RNA was extracted from pools of 10 pteropods with 1 pool for each of the replicate treatment tanks (*n* = 3). This sampling scheme provided 3 biological replicate pools for each treatment.

Total RNA extractions were performed using 500 μl of Trizol**®** reagent according to the manufacturer’s instructions (Invitrogen). Each sample of total RNA was analyzed for quantity and purity using the NanoDrop**®** ND100. RNA integrity was assessed by electrophoresis using the TapeStation 2200 system (Agilent Technologies) to calculate RNA integrity (RINe) scores. Only samples with RINe scores above 8.5 were used for library preparation in order to avoid sequencing degraded RNA products. Here, RINe scores are similar to the Bioanalzyer RIN score. Both metrics are an assessment of RNA degradation calculated by running the sample on a mini-capillary gel, and measuring the concentration of degraded RNA in relation to the intensity of the 18S ribosomal peak [29].

Separate libraries (*n* = 39) were generated from high-quality RNA using the Illumina TruSeq Stranded mRNA HT Sample Preparation Kit. Briefly, 2 μg total RNA was added to Illumina RNA Purification beads to isolate mRNA from the total RNA. Purified mRNA was then enzymatically fragmented into 300 base-pair (bp) fragments and reverse transcribed into cDNA. Second strand cDNA synthesis utilized the dUTP method, which creates a strand-specific cDNA library [30]. Following second strand synthesis, cDNA fragments were size selected using AmpureXP® beads (Beckman Coulter), and the library was amplified with 12 cycles of PCR according to the manufacturer’s instructions. Completed libraries were checked for mean lengths of 428 bp (300 bp insert +128 bp sequencing adapters) using the Agilent TapeStation. Concentrations of each final library was quantified using a Qubit**®** 3.0 flourometer (Life Technologies). All libraries were sequenced in two pools, each on two HiSeq4000 lanes. Sequencing was performed at the UC Davis Genome Center on an Illumina HiSeq4000 sequencer using paired-end 100 base-pair sequences. Outputs for each lane were treated independently throughout the quality control steps after which technical replicate samples were merged.

### Mapping and identification of differentially expressed genes

**Fig. 1:**
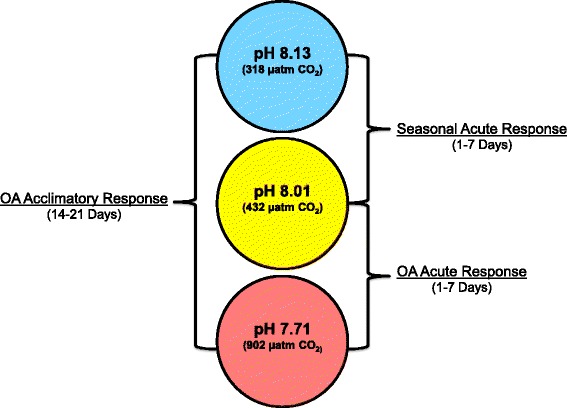
Gene Expression Comparisons. Gene expression comparisons were designed to assess acute and acclimatory responses to 3 scenarios: (i) Seasonal acute response, (ii) Seasonal acclimatory response, and (iii) a future ocean acidification response

Raw reads from each sample were processed using Trimmomatic to remove sequencing adapters and bases with a PHRED33 score < 20 [27]. Only trimmed sequences that were ≥75 bp were included in downstream analysis. Trimmed reads of each sample were used to improve the previously published de novo transcriptome [31]. Briefly, trimmed sequence reads were assembled into a de novo transcriptome using the assembler Trinity (v2.3.2). The assembled contigs were then annotated using the NCBI software blast + (version 2.3.0) to execute a blast-x search (e-value ≤1e ^−5^) against the previously published *L. h. antarctica* transcriptome [31], the NCBI non-redundant protein database and the reference genome for *Aplysia californica* (NCBI assembly GCF_000002075.1). Trimmed reads were mapped to the de novo transcriptome using RSEM with default parameters [32]. Read counts from each lane were merged for each sample immediately prior to the analysis of differential expression. Differential expression of transcripts was evaluated using a negative binomial general linearized model (GLM) as integrated in the Bioconductor R package edgeR-robust [33]. Within edgeR, we defined up-regulated transcripts as having a log_10_ fold change ≥1, and down-regulated transcripts as having a log_10_ fold change ≤ −1 with a false discovery rate (FDR) ≤ 0.05 [34]. Differential expression was further assessed using three major comparisons: (i) using days 1 and 7, short-term, acute responses to seasonally relevant changes in *p*CO_2_, (ii) acute responses to ocean acidification again using data from day 1 and day 7, and (iii) the longer-term, acclimatory response to ocean acidification conditions using samples from days 14 and 21 (Fig. 1). From these comparisons, gene ontology enrichment analysis was performed using the Fisher’s exact test (FDR ≤ 0.05) implemented in the Blast2GO software (version 2.6.0) [35].

Finally, we examined expression profiles for sets of genes involved in four physiological and cellular processes: (i) genes involved in shell formation, (ii) genes associated with the cellular stress response, (iii) genes associated with metabolism, and (iv) genes associated with neural function. Gene identifiers for shell formation, metabolism and the cellular stress response were collected from the published Pacific oyster (*Crassostrea gigas*) genome [36]. Gene identifiers for neural function were retrieved from the published *Limacina inflanta* transcriptome, and hits for all physiological groups were blasted against the *L. h. antarctica* master transcriptome. Expression levels were calculated with RSEM and expressed as fragments per kilobase of transcript per million mapped reads (FPKM). FPKM values were then used to generate expression profile heatmaps for each process at both acute and acclimatory response times. Genes with similar Z-scores for each treatment across sampling days were further assessed for enrichment against the annotated transcriptome using the Fisher’s exact test (FDR > 0.05).

## Results

### Experimental pCO_2_ exposures

During the *p*CO_2_ exposure experiment, treatment conditions throughout the 21-day period were stable (Table 1). In addition, mortality in the experiment as a whole was low with fewer than 20 mortalities for any single tank across the 21-day experiment (data not shown). Throughout the exposure period, actively swimming individuals were consistently present and only these individuals were sampled for analysis.

### Transcriptome assembly and annotation

Sequencing of the 39 libraries was completed on 2 lanes of an Illumina HiSeq4000 yielding a total of 803 million reads (~20.6 +/− 4.1 million reads per library, range: 15.4–24.7 million reads per library). Quality and adapter trimming removed approximately 3% of the reads. All remaining high-quality reads from each of the 39 libraries were used to generate a de novo transcriptome using Trinity (v.2.3.2). Trinity produced a total of 403,210 contigs; RSEM was implemented to map sequence reads to the de novo transcriptome, which resulted in an average of 71% of sequences being successfully mapped (mapping range: 67.5% - 73.8%). The Trinity contigs were subsequently filtered to only include sequences that were longer then 200 bp and had at least 1 count per million in 9 of the 39 samples. This filtering resulted in 83,211 expressed contigs with ~13.4 +/− 3.0 million reads per sample (range: 9.4–23.0 million reads per library). A total of 37,517 (45.1%) of these expressed contigs were successfully annotated using a Blastx search against the previously published *L. h. antarctica* transcriptome, the NCBI non-redundant database, and the Swissprot database. Annotations were combined with sequence reads, gene ontologies, and KEGG pathway identifiers using the Blast2GO software (version 2.6.0) [35]. Additional transcriptome assembly statistics are available in Additional file 1.

### Analysis of differential gene expression

Differential gene expression was assessed for multiple comparisons using the time when the pteropods were sampled from the Z-system tanks, and in terms of what pH treatments were compared. The results are described below and summarized in Table 2. In addition, transcripts named in the differential expression analysis, and in the analysis for enrichment, are listed in Additional files 2, 3, 4 and 5.

**Table 2:** Pair-wise differential expression results

pH comparisons	Day	Up-regulated transcripts	Down-regulated transcripts
8.13 vs 8.01	1	168	95
	7	624	1554
7.71 vs 8.01	1	247	1818
	7	22	18
7.71 vs. 8.13	14	447	1197
	21	299	873

#### Pair-wise gene expression analysis

##### DE following acute exposure to pH treatments

Here we report changes in the transcriptome after exposure to pH conditions for 1 day (d1) and again after 7 days (d7). These comparisons represent two scenarios that assess differential gene expression in response to (1) seasonal changes in pH that occur in the present-day (pH 8.13 vs. 8.01), and (2) future ocean pH conditions (pH 7.71 vs. 8.01). The first comparison of pH 8.13 vs. 8.01 represents gene expression patterns that might occur in response to ecologically relevant conditions, but on an acute time scale, i.e., on the order of days and not on the actual months-long time scale of the real-time transition of austral winter to austral summer. This sampling regime was necessitated by the reality of working in the field in Antarctica where the time to conduct experiments is limited.

Acute exposure to seasonally relevant changes in pH induced measurable changes in gene expression levels with a total of 263 transcripts changing in directional expression. Specifically, 168 transcripts were up-regulated, and 95 transcripts down-regulated in the high-pH (8.13) treatment when compared to the mid-pH (8.01) treatment (Table 2). The up-regulated transcripts were involved in methyltransferase activity, DNA Binding, ATPase activity, oxidoreductase activity, ion binding, and transmembrane transporter activity (Additional file 2). Further examination of this differential gene expression pattern found that within the up-regulated group there was enrichment of 5 gene ontologies associated with cytoskeleton function, calcium ion binding and microtubule based movement (Additional file 4, FDR <0.01). In contrast, the 95 down-regulated transcripts were involved in DNA and poly(A) RNA binding, helicase activity, and the cytoskeleton structure; however, these transcripts had no associated enrichment patterns (Additional file 2).

By day 7, there was a significant increase in differential expression with approximately 8-times more genes changing in expression. Specifically, we identified 1554 transcripts up-regulated and 624 transcripts down-regulated in the high-pH (8.13) treatment when compared to the mid-pH (8.01) treatment (Table 2). Among these up-regulated transcripts 31 sequences were involved in anatomical structure development, 123 were involved in cytoskeleton structure, and 70 were involved in ion binding (Additional file 2). Enrichment analyses of these differentially expressed transcripts identified 34 enriched gene ontologies that included calcium ion binding, actin binding, calmodulin binding, and the proteinaceous extracellular matrix (Additional file 4, FDR < 0.01). Within the 624 down-regulated transcripts, 28 were involved in catalytic activity, 17 were involved in metabolic processes, and 29 of sequences were involved in cellular protein modification (Additional file 2). Once again, while there were a large number of differentially expressed transcripts that were down-regulated, there were no enrichment patterns associated with these transcripts.

The second comparison at d1 where future low pH conditions were examined (pH 7.71 vs. 8.01) identified a more pronounced change in the transcriptome. Here, 247 transcripts were up-regulated and 1818 transcripts were down-regulated in the low-pH (7.71) treatment when compared to the mid-pH (8.01) treatment (Table 2). Of the up-regulated transcripts, 15 were involved in ion binding, 14 were involved in catalytic activity, and 15 sequences were involved in catabolic processes (Additional file 3). However, a fisher’s exact test of the 246 up-regulated transcripts produced no significant enrichments (FDR <0.05). Within the 1818 down-regulated transcripts, 152 transcripts were involved in catalytic activity (hydrolase and oxidoreductase activity), 62 sequences were involved in ion binding, and 101 sequences were involved in combined metabolic processes (Additional file 3). Enrichment analysis of the 1813 down-regulated transcripts produced significant enrichments (FDR <0.01) in 20 ontological categories, including the proteinaceous extracellular matrix, transporter activity, catalase activity, chitin binding, and lipid transport (Additional file 5).

By d7, exposure to the low pH condition tapered to include 22 up-regulated transcripts, and 18 down regulated transcripts. Up-regulated transcripts were involved in transmembrane transport, the nuclear pore complex, and mRNA processing (Additional file 3). The set of down-regulated transcripts contained transcripts that code for structural constituents of ribosomes, and developmental processes. Across both up- and down-regulated transcripts there was no gene ontology enrichment.

##### Changes in the transcriptome after 14 and 21 days

In order to assess changes in the transcriptome after long-term acclimation to the pH treatments, we measured DE after 14 days (d14) and 21 days (d21) of incubation in treatment conditions. For these longer-term exposures, only the end-member pH treatments (pH 7.71 vs. pH 8.13) were compared. This comparison spans the in situ pH range that pteropods are expected to experience annually by the year 2050 [9].

Following 14 days of exposure to pH 7.71, we observed a pronounced transcriptomic response where 377 transcripts were up-regulated, and 1197 transcripts were down-regulated (Table 2). Among the up-regulated transcripts, 32 transcripts were involved in cellular protein modification, 50 transcripts were involved in metabolic processes, and 36 transcripts were involved in macromolecule biosynthesis (Additional file 3). Enrichment analysis yielded no enriched gene ontologies for the up-regulated transcript set. Among the 1197 down-regulated transcripts a total of 94 transcripts were associated with metabolic processes, 105 with ion binding, and 124 with catalytic activity (Additional file 3). Further defining these terms found 75 transcripts were associated with calcium ion binding, 49 transcripts were associated with the cytoskeleton, and 21 transcripts were associated with ubiquitination. Enrichment analysis of the down-regulated transcripts identified 26 gene ontologies including single organism cellular process, ATP binding, calcium ion binding, protein kinase activity, and ion transmembrane transport (Additional file 5, FDR < 0.01).

After 21 days of exposure to pH 7.71, the response was somewhat attenuated with 241 up-regulated transcripts, and 872 down-regulated transcripts (Table 2). Among the up-regulated transcripts 24 transcripts were involved in translation, 17 transcripts were structural components of ribosomes, and 24 transcripts were involved in the formation of membrane-bound organelles (Additional file 3). In addition, 16 transcripts were found to be involved with catalytic activity, 20 with metabolic processes, and 6 with ion binding. Enrichment analysis of these up-regulated transcripts identified 7 enriched gene ontological terms (Additional file 5, FDR <0.05), all associated with membrane biogenesis and assembly. Among the down-regulated transcripts, 77 transcripts were associated with catalytic processes, 57 with metabolic processes, and 59 with ion binding (Additional file 3). A further definition of these terms found 40 transcripts associated with calcium ion binding, and 16 transcripts associated with the cytoskeleton. Enrichment analysis identified enrichment in 9 gene ontologies associated with ATP binding, ATPase activity, microtubule motor activity, and transport (i.e. ATP-binding cassette sub-family A) (Additional file 5, FDR < 0.01).

#### Physiologically relevant gene expression analysis

In order to better characterize the response of the pteropods to *p*CO_2_ exposure, physiologically relevant gene sets were identified with Blastx searches of the *L . h. antarctica* transcriptome against the *C. gigas* genome [35] and the pteropod *Heliconoides inflatus* transcriptome [37]. These genes were used to identify differential expression patterns for four major physiological processes: shell formation, metabolism, the cellular stress response (CSR), and neural signaling. For each process we analyzed expression profiles for the acute (T_0_, d1, and d7), and acclimatory (d14 and d21) time points. The analysis of differentially expressed genes using the Fisher’s exact test revealed enrichment only within the metabolic physiological process. Gene names from this analysis are listed in Additional files 6, 7, 8 and 9.

##### Shell formation response

**Fig. 2:**
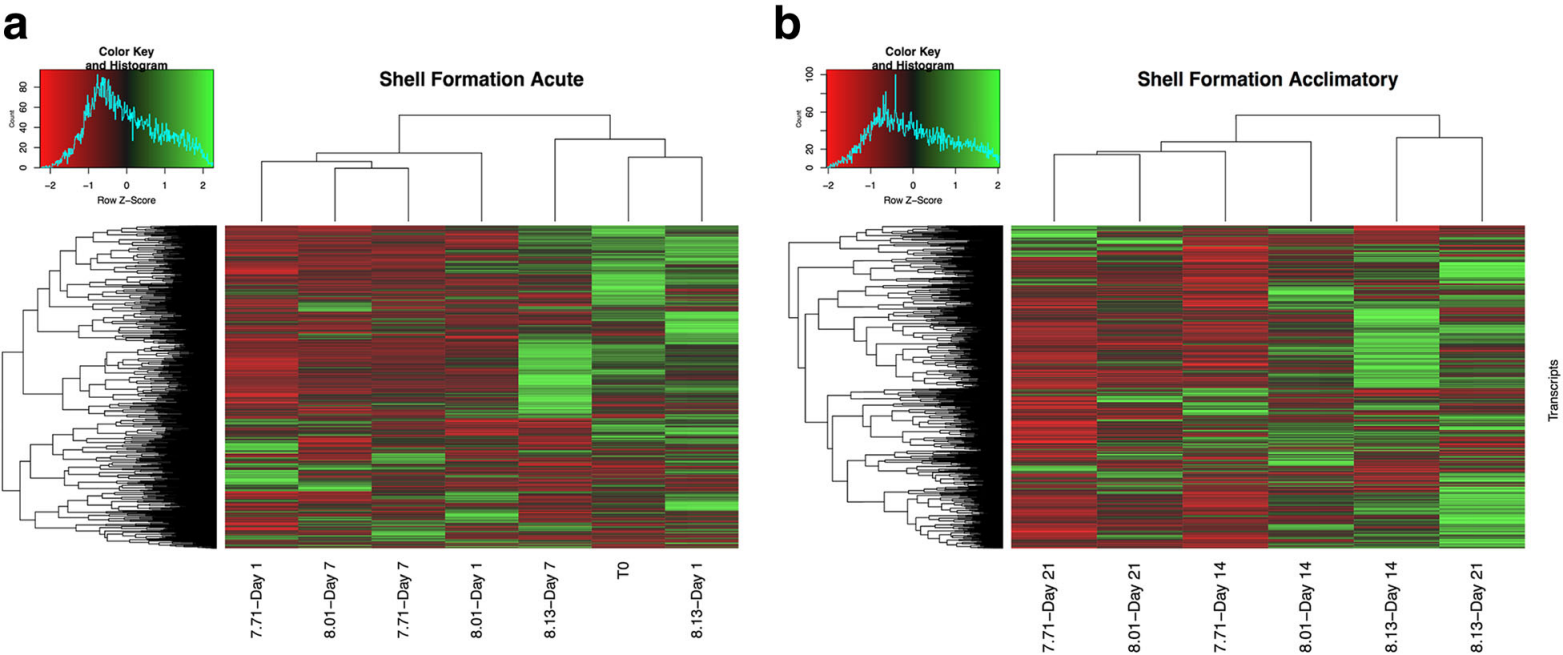
Shell formation genes expression profiles. Heatmap of genes associated with shell formation. **a** clustering and heatmap of Z-scores for T_0_ and the acute response (d1 and d7) to all 3 treatment conditions (**b**) clustering and heatmap of Z-scores for the acclimatory response (d14 and d21). For both **a** and **b** are identified as treatment (pH 8.13, pH 8.01, pH 7.7) and Day (1,7,14,21)

The Blastx search of the *L. h. antarctica* transcriptome against genes related to shell formation in the Pacific oyster [35] identified 1497 shell formation transcripts. Expression z-scores were calculated from FPKM values and filtered to include only these transcripts (Additional file 6). Expression profiles were analyzed at both the acute and acclimatory time points (Fig. 2ab).

The acute response within this subset of transcripts showed a clear clustering of the high-pH treatment (pH 8.13) with T_0_ for both d1 and d7 (Fig. 2a). This cluster identified 1015 up-regulated, and 482 down-regulated shell formation transcripts. The mid- and low-pH treatments (8.01 and 7.71, respectively) cluster in a separate group with d7 showing the highest levels of similarity with 574 up-regulated, and 923 down-regulated shell formation transcripts (Additional file 6).

This pattern was accentuated in the acclimatory response (d14 and d21) where higher expression levels of shell formation genes were detected in the high-pH treatment than in either the mid- or low-pH treatments (Fig. 2b). In addition, at these time points, treatments clustered together, showing a treatment-specific response. Specifically within the high-pH treatment, we identified 805 up-regulated, and 692 down-regulated shell formation transcripts. Within the mid-pH treatment these tapered to 530 up-regulated, and 967 down-regulated transcripts. This decline in expression continued in the low-pH treatment where only 282 transcripts were up-regulated and 1215 were down-regulated (Additional file 6).

##### Cellular stress response

**Fig. 3:**
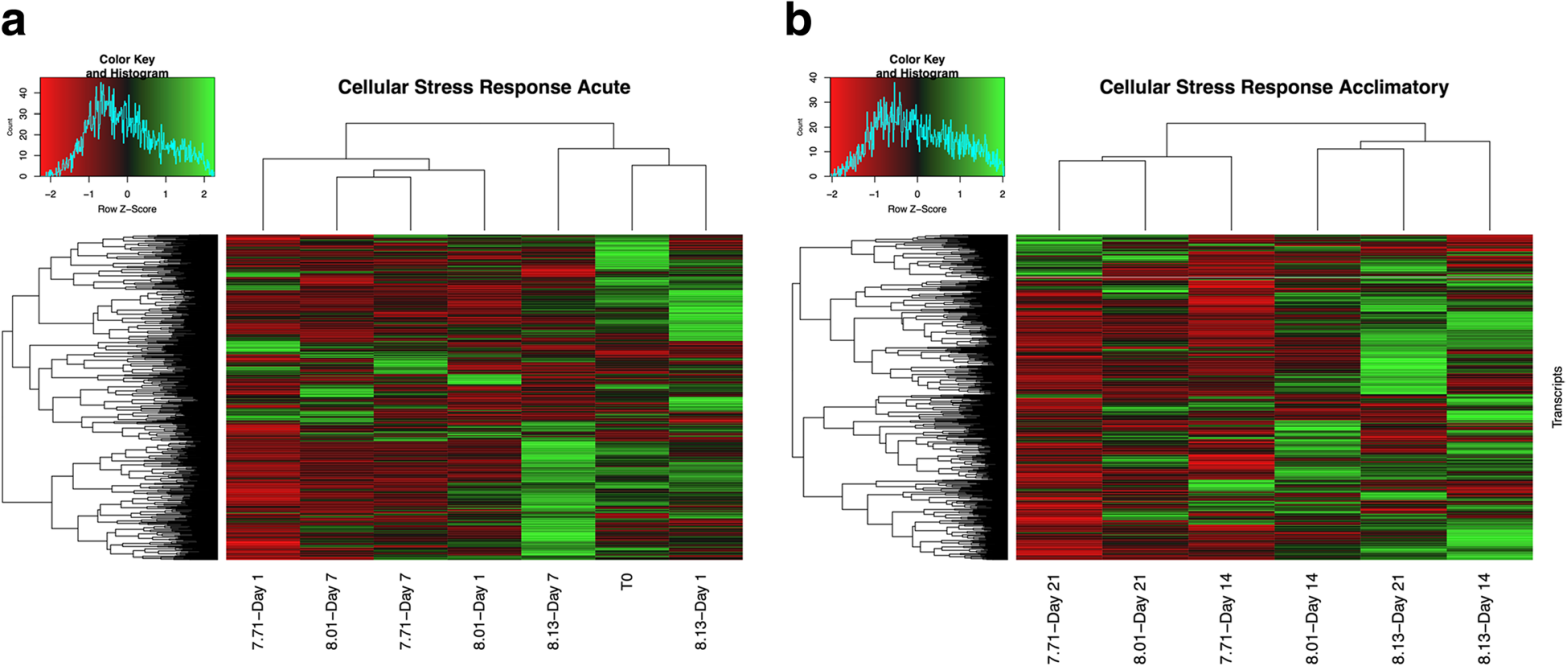
Cellular stress response genes expression profiles. Heatmap of genes associated with the cellular stress response. **a** clustering and heatmap of Z-scores for T_0_ and the acute response (d1 and d7) to all 3 treatment conditions. **b** clustering and heatmap of Z-scores for the acclimatory response (d14 and d21)

The Blastx search of the *L. h. antarctica* transcriptome against known stress-related genes from the Pacific oyster [35] identified 695 CSR transcripts. Expression FPKM values were filtered and z-scores calculated for these 695 CSR transcripts (Additional file 7). Expression profiles were then analyzed at both the acute and acclimatory time points (Fig. 3a, b).

The acute response within this subset of transcripts revealed expression patterns that grouped T_0_, d1 of the high-pH (8.13) exposure, and d7 of the mid-pH (8.01) treatments (Fig. 3a). Differential expression analysis for the CSR transcripts at these stages revealed T_0_ to have 481 up-regulated transcripts and 214 down-regulated transcripts and the high-pH treatment (d1 and d7) to have an average of 466 up-regulated and 229 down-regulated transcripts. This clearly contrasts with the mid- and low-pH treatments that had on average of 272 up-regulated and 423 down-regulated CSR transcripts (Additional file 7).

The acclimatory response displayed a different trend wherein the mid-pH treatment clustered with the high-pH treatments on d14, but clustered with the low-pH treatment on d21 (Fig. 3b). Assessing expression levels for these patterns revealed that the high-pH treatment on average (d14 and d21) expressed 371 up-regulated, and 324 down-regulated transcripts, the mid-pH treatment on average expressed 259 up-regulated, and 436 down-regulated transcripts, and the low-pH treatment on average expressed 130 up-regulated and 565 down-regulated CSR transcripts (Additional file 7).

##### Metabolic response

**Fig. 4:**
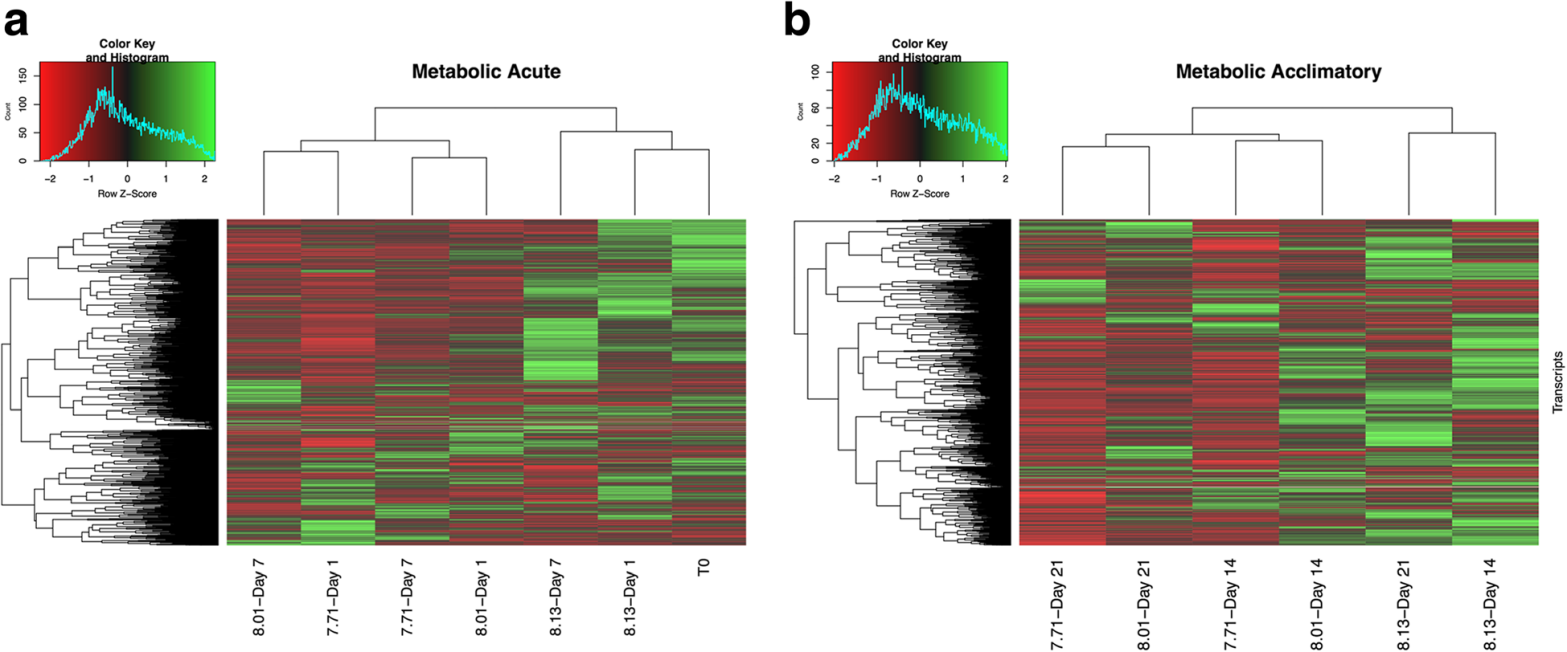
Metabolic genes expression profiles. Heatmap of genes associated with metabolism. **a** clustering and heatmap of Z-scores for T_0_ and the acute response (d1 and d7) to all 3 treatment conditions. **b** clustering and heatmap of Z-scores for the acclimatory response (d14 and d21)

The Blastx search of the *L. h. antarctica* transcriptome against known metabolic genes from the Pacific oyster [35] identified 2139 metabolic transcripts. Expression FPKM values were filtered to include these metabolic transcripts, and z-score and expression profiles were again analyzed at both the acute and acclimatory time points (Fig. 4a,b, Additional file 8).

Clustering of the metabolic transcripts at the acute time points once again grouped T_0_ with the high-pH treatments (d1 and d7) (Fig. 4a). In addition, the mid-pH and low-pH treatments were very closely associated w the mid-pH d1 clustered with d7 of the low-pH treatment and d7 of the mid-pH treatment clustered with d1 of the low-pH treatment. The metabolic genes at this acute exposure once again are the most highly expressed in the high-pH treatment with an average of 1362 transcripts up-regulated for both d1 and d7. In contrast, the mid- and low-pH treatments had an average of 816 up-regulated metabolic transcripts between d1 and d7 (Additional file 8).

The acclimatory response of this subset of metabolic transcripts revealed both a treatment-specific response, where the high-pH maintained the highest expression level of transcripts, and a temporal response where the mid- and low-pH treatments clustered together by day 21 (Fig. 4b). Evaluating the transcripts associated with each treatment revealed that the high-pH treatment once again had the highest level of expression with an average (d14 and d21) of 1082 up-regulated metabolic transcripts, the mid-pH treatment had 737 up-regulated metabolic transcripts, and the low-pH treatment only had 476 up-regulated metabolic transcripts (Additional file 8).

To further assess the metabolism-associate gene expression a Fisher’s exact test for each treatment revealed over-expression of genes for both the mid-pH and high-pH treatments. Enrichment within the up-regulated transcripts in the mid-pH treatment identified over-expression of genes that are associated with calcium ion binding. In addition, this analysis identified an under-expression of genes associated with oxidoreductase activity. Enrichment analysis within the up-regulated transcripts in the high-pH treatment identified enrichment of 3 gene categories: nucleolus, rRNA metabolism, and histone modification. Finally, analysis of the down-regulated transcripts in the high-pH treatment identified enrichment of calcium ion binding, transmembrane signaling receptor activity, and sulfuric ester hydrolase activity. In addition, there were 10 gene categories under-represented among the down-regulated transcripts, these include genes involved in RNA metabolic processes, translation, and intracellular membrane-bound organelle cellular components (Additional file 9).

##### Neural response

**Fig. 5:**
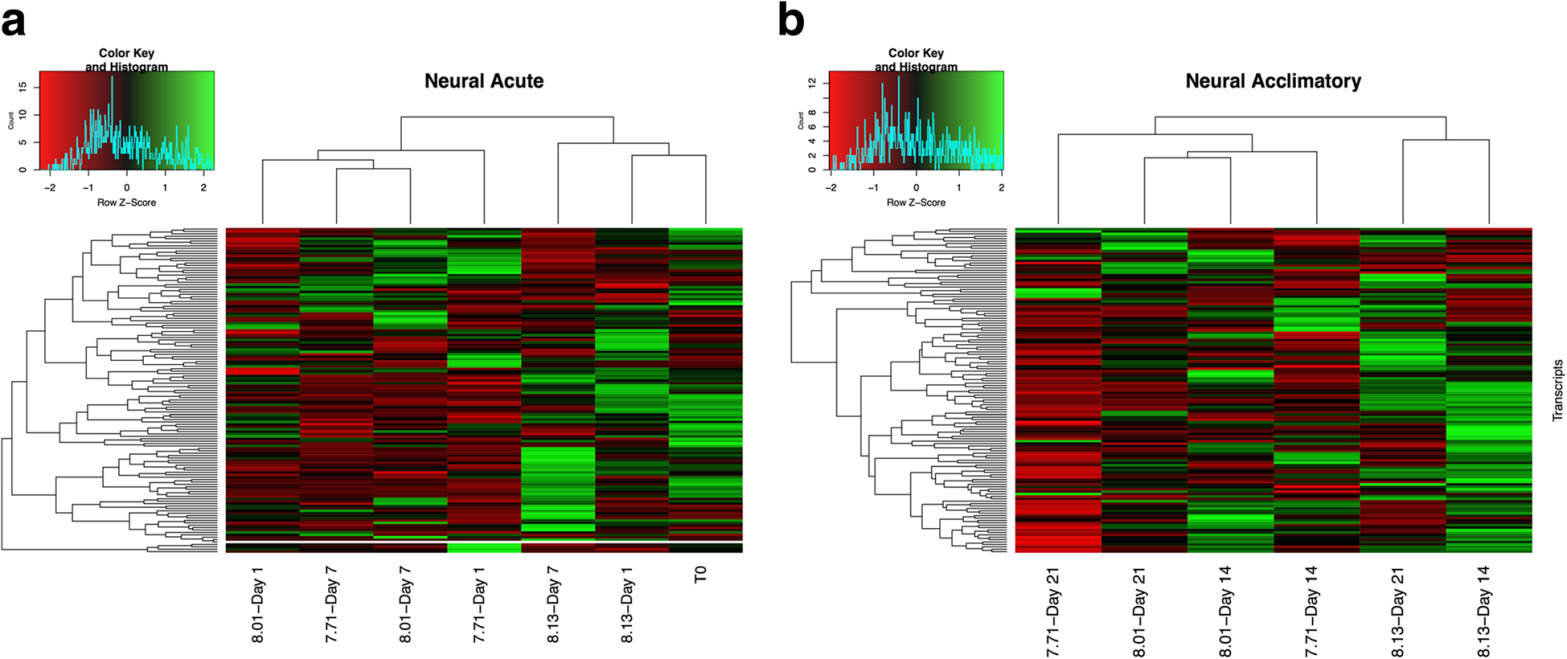
Neural genes expression profiles. Heatmap of genes associated with neural function. **a** clustering and heatmap of Z-scores for T_0_ and the acute response (d1 and d7) to all 3 treatment conditions. **b** clustering and heatmap of Z-scores for the acclimatory response (d14 and d21)

The Blastx search of the *L. h. antarctica* transcriptome against previously identified neural genes differentially expressed in the pteropod *Heliconoides inflatus* [37] identified 191 neural transcripts. Expression FPKM values were filtered and z-scores calculated for these 135 neural transcripts (Additional file 9). Expression profiles for neural transcripts revealed large patterns associated with both acute and acclimatory responses to the pH treatments (Fig. 5a,b).

Clustering of the neural transcripts at the acute time points once again revealed clustering of high expression levels in both T_0_ and the high-pH treatments (d1 and d7) while the mid- and low-pH treatments exhibited dampened expression levels (Fig. 5a). Specifically, the high-pH treatment had an average of 79 up-regulated neural transcripts while the mid- and low-pH treatments had 61 and 55 up-regulated neural transcripts, respectively (Additional file 9).

This trend of higher expression levels for genes related to neural processes in the high-pH treatment was maintained in the acclimatory time points (Fig. 5b). At these time points, we observed an average of 68 up-regulated transcripts in the high-pH treatment, 47 up-regulated transcripts in the mid-pH treatment, and an average of 33 up-regulated transcripts in the low-pH treatment. Expression levels continued to decline in the low-pH treatment such that by day 21, only 23 neural transcripts were observed to be up-regulated (Additional file 9).

## Discussion

Changes in the transcriptome can serve as a metric of physiological plasticity in metazoans in response to changes in their abiotic environment [1, 4, 6, 7, 38]. The goal of this study was to assess patterns of differential gene expression in the polar pteropod, *Limacina helicina antarctica*, in response to pH conditions that reflect both present-day, seasonal variability in pH, and pH conditions predicted for future ocean acidification. In general, we found that the *L. h. antarctica* transcriptome changed in response to pH treatments that represented pH values for both seasonal, and ocean acidification conditions. In addition, the *L. h. antarctica* transcriptome changed as a function of time of exposure to the various pH treatments used in the experiment. Lastly, the expression patterns observed involved genes found in key processes that would be physiologically significant to a calcifying marine mollusk under these abiotic conditions in that these conditions may challenge biogenic calcification and energy allocation in a juvenile marine calcifier. Specifically, these four groups of genes are those involved in shell formation, the cellular stress response, metabolism, and neural function.

### Differential gene expression analysis

#### Acute transcriptomic responses

We found that *L. h. antarctica* had a robust transcriptional response to acute pH changes that represent present-day seasonal pH values (Tables 1 and 2). Specifically, in comparisons of expression for pH 8.13 to 8.01 at day 1, strong up-regulation of transcripts involved with large-scale cellular function indicated a regulatory response to more alkaline conditions that typifies the transition from winter into summer [12]. Changes in transcript abundance of genes associated with DNA binding and epigenetic modifications such as DNA methylation (e.g. methyltransferases) indicated that the response may have involved epigenomic modifications that could act as mechanisms to change the transcriptome (Additional file 2).

Along with these genomic/epigenomic changes, we also observed increases in transcripts for calcium binding proteins (e.g. calmodulin-like proteins), ion binding, and transmembrane transporters, all genes known to be important in biogenic calcification (Additional file 2). In addition, functional enrichment analysis revealed enrichment of transcripts involved with cytoskeleton function and calcium ion binding (Additional file 4). The up-regulation of these gene groups in the high-pH treatment suggests pteropods increased shell formation when held in conditions highly favorable for calcification.

Together, these results provide evidence that exposure to high pH conditions, which are presumably not physiologically stressful, led to broad scale transcriptomic changes that are focused on genome reorganization, and the maintenance and synthesis of cytoskeletal and calcified structures. Following 7 days of exposure these trends further intensified with 1554 up-regulated transcripts that expanded the d1 regulated set to include anatomical structure development, and enrichment of cytoskeletal protein binding (Additional file 2). The continued increase in expression of genes associated with both the cytoskeleton and calcification indicated that pteropods up-regulate gene pathways associated with enhancing biogenic calcification when exposed to high-pH (8.13) conditions, the pH value observed during the peak of summer.

An examination of the suppressed transcripts for the acute seasonal pH treatment revealed minor down-regulation in three gene types: nucleotide binding, helicase activity, and cytoskeleton structure (Additional file 2). While these results are similar to the transcripts identified in the up-regulated category, they represent a small number of isoforms that may have additional functions within these polar gastropods. Indeed, after 7 days of exposure, these contrasting transcripts were no longer present, and the residual suppressed transcripts were primarily involved in protein degradation (Additional file 2).

In general, differential gene expression analysis in juvenile *L. h. antarctica* revealed that acute exposure to pH 8.13 produced dramatic transcriptional changes that were focused on cytoskeletal development and genome organization. While a robust transcriptional response is consistent with previous studies of the arctic form of *L. h. helicina* and the Mediterranean pteropod *Heliconoides inflatus* [22, 37], our results are the first to characterize the effects of pH on the Antarctic form of *L. helicina.* In addition, these data suggest that the juvenile pteropods were responding positively to seawater conditions that are typical of summer, a time when food supply is high and the growth potential is presumably also optimal for this polar species.

In contrast, exposure to pH conditions of ocean acidification (pH 7.71) resulted in suppression of calcification, lipid transport, and metabolic function; suggesting that these conditions are physiologically stressful (Additional file 3). During the first 24 h of exposure, the dominant signature was a large-scale down-regulation of transcripts with enrichment in 20 gene ontologies (Additional file 5). Among these gene ontologies, three are noteworthy with regard to this suppression of gene expression in *Limacina*: (1) calcium ion binding, (2) chitin binding, and (3) the proteinaceous extra-cellular matrix. Within these down-regulated ontological groups are transcripts that code for tyrosinase, multiple chitin-binding proteins, calmodulin, carbonic anhydrase, Von Willebrand factor type A, Sushi, and bone morphogenic protein 1-like (Additional file 3). These genes are all known to have fundamental roles in the formation of the calcium carbonate shell of mollusks [39–43]. The suppression of these transcripts after 24 h of exposure to low pH conditions indicates that pH-stressed pteropods rapidly suppress biogenic calcification.

In addition to changes in genes related to calcification, we also found significant impacts on genes associated with lipid transport, suggesting a shift in energetic needs in response to conditions of ocean acidification (Additional file 3) [44]. The down-regulation of lipid transport genes points to a global disruption of energy allocation that corroborates other observations in studies on marine invertebrates including oysters, Antarctic krill and corals [45–47]. Changes in energy allocation for *L. h. antarctica* have been previously reported, wherein energy reserves in eggs were decreased when adults were exposed to low-pH conditions [48]. Similar effects of pH have also been reported in green sea urchins [49] and copepods, where low pH resulted in significant impacts on energy allocation and egg production [50].

That exposure to low-pH conditions impacted the energy budget of *L. h. antarctica* was further underscored by the down-regulation of metabolically important transcripts such as carbonic anhydrase, glucose dehydrogenase and malate dehydrogenase (Additional file 3). These genes are central to ATP production in metabolic pathways, and the integrity of energy metabolism has been identified as a key indicator of tolerance to abiotic stress [44]. Specifically, down-regulation of glucose and malate dehydrogenase indicates a reduced ability to produce NADPH, a critical cofactor involved in oxidation-reduction, glutathione generation, and lipid synthesis. Down-regulation of these genes in response to conditions of ocean acidification has been documented in corals, oysters, and the great spider crab [45, 51, 52].

By day 7, differential expression between the mid-pH (8.01) and the low-pH (7.71) was reduced to a total of 40 differentially expressed transcripts (Table 2). The up-regulated transcripts were concentrated around transcription-related processes while down-regulated transcripts were involved in developmental processes. With so few differentially expressed transcripts, no gene ontological enrichment was present. The shift from high numbers of differentially expressed genes to this muted response suggests the transcriptomic profiles for the mid- and low-pH treatments were converging, an observation further supported by gene expression analysis of genes involved in physiological and cellular processes.

#### Differential expression following short-term acclimation exposure

As was observed with the acute exposures of juvenile *Limacina*, longer-term acclimation (14 to 21 days) to low pH conditions resulted in significant changes in gene expression (Table 2). In general, gene expression varied from the short-term response in that we observed a higher percentage of down-regulated transcripts at both d14 and d21. Specifically, at d14 the largest signal was among down-regulated transcripts with enrichment in 26 gene ontologies, an indication of large-scale reduction in transcription (Additional file 6). This was further supported by down-regulation of metabolic and ion binding transcripts, and indicated significant suppression of genes involved in biogenic calcification similar to the differential gene expression patterns observed in the acute response (Additional file 3). This pattern continued into d21 with down-regulation of cytoskeleton and calcium ion binding transcripts. Enrichment at d21 identified 9 enriched gene ontologies that reflect patterns identified during the acute exposure to low pH conditions (Additional file 6). With 872 down-regulated transcripts that continued to span calcium ion binding, metabolic function and cytoskeleton development, this pattern of differential expression is evidence that present-day pteropods genotypes do not modulate expression of candidate calcification genes that may be critical to continued calcification during the under-saturated austral winter conditions forecast for the Southern Ocean.

### Analysis of differential gene expression in physiologically relevant processes

In order to better characterize the effect of pH on *L. h. antarctica,* we focused on 4 major processes that are key to pteropod physiology: shell formation, metabolism, the cellular stress response, and neural function. Overall, the differential gene expression analysis found that all 4 of these groups were predominately down-regulated when pteropods were exposed to low pH conditions. Below we briefly discuss each set of transcripts in these 4 categories.

#### Shell formation response

Expression patterns for genes involved in shell formation at the acute time points (d1 and d7) revealed a pattern of increased expression under the high-pH condition of 8.13, and very close clustering of expression profiles for both the mid-pH and low-pH conditions (Fig. 2a,b). At the acclimatory time points (d14 and d21), expression patterns for the three treatments partitioned into 3 categories that were associated with treatment conditions: high expression (pH 8.13), mid expression (pH 8.01), and low expression (pH 7.71) (Fig. 2b). These results support our position that long-term exposure to under-saturated conditions, mimicking those that *L. h. antarctica* are predicated to experience in winter by the year 2050, could limit the ability for pteropods to maintain calcified structures, and highlights the necessity for further functional validation of these effects.

Previous gene expression analysis for calcifying marine invertebrates has focused on a subset of shell formation genes that are impacted by changes in pH including calmodulin-like protein, chitin synthases, C-type lectines, perlucin, and collagen associated transcripts [2, 22, 37, 53]. Within this subset of shell formation related transcripts, there was a clear down-regulation associated with exposure to the low pH treatment (Additional file 6). Among these, calmodulin-like transcripts have been identified as important for calcification in the pearl oyster [42], and have been shown to be down-regulated in response to low pH in both oysters and corals [41, 53]. Chitin synthase is also critical in coordinating shell formation for mollusks [54], and has also been reported to be down-regulated in *Limacina spp.* [22]. Finally, collagen-related transcripts (including C-type lectine, perlucin, and collagen-associated transcripts) have been shown to be important in shell formation in the disk abalone (*Haliotis discus discus*), and have been reported to be highly abundant in the mantle tissue of the Mediterranean mussel (*Myilus galloprovincialis*) [55, 56].

The heatmap displaying the broad patterns of gene expression in these shell formation genes (Fig. 2a, b) highlighted a subset of genes that were activated in response to low pH. Among these up-regulated transcripts were tyrosinase tyr-3, chromatin assembly factor 1 subunit B-like, ribosome biogenesis homolog, ribosome-releasing factor mitochondrial-like, and a RNA-directed DNA polymerase from mobile element jockey-like gene (Additional file 6). Among these, tyrosinase tyr-3 has previously been shown to be up-regulated in the biomineralization process in the blue mussel (*Mytlius edulis*), and is associated with formation of new periostracum [57, 58].

#### Metabolic response

Expression patterns assessed in the metabolic response category revealed both temporal and pH-related patterns (Fig. 3a,b). Among these metabolic transcripts the highest levels of expression observed were in the T_0_ samples, and in the high pH treatments (Fig. 3a,b). The maintenance of T_0_ levels of expression for the first week of exposure, and continued grouping of the high pH treatments, suggests that food limitation was not a major obstacle for pteropods held in the pH 8.13 treatment. The mid and low-pH treatments however showed significant depression of metabolism-associated transcripts that grouped the low and mid pH treatments with time of exposure. This suppression of metabolic genes in response to low pH has been documented in corals, urchins, and the Mediterranean pteropod *Heliconoides inflautus* [2, 37, 59, 60]. The enrichment of genes in both the mid-pH and high-pH treatments along with a dramatic reduction in metabolic transcript expression suggests energy demands under short-term exposure to low pH conditions were not met by an increase in metabolism. This result may reflect the limited feeding opportunities experienced in our flow-through Z-system; however, in situ, little is known about food availability throughout the light-limited austral winter.

#### Cellular stress response

Differential expression patterns for genes involved in the CSR provided additional evidence of a compromised transcriptional response in *L. h. antarctica* in response to low pH. Up-regulated CSR transcripts associated with exposure to the low pH treatment were: HSP 70, heat-shock factor 2 binding protein-like gene, hydroxysteroid dehydrogenase 2-like gene, and a short-chain dehydrogenase/reductase family member 11-like gene (Additional file 6). In contrast, the transcripts down-regulated in the low pH conditions included those coding for multiple components of the cytochrome p450 pathway, the universal stress A-like protein, HSP 90-beta, and a glutathione mitochondrial-like protein.

The differential expression of key chaperones such as HSP 70 and HSP 90, genes critical for maintaining protein structure/function during oxidative stress, indicates that pH exposure in polar waters does not illicit a classic cellular stress response. Rather, exposure to low-pH conditions led to slight differential expression of these constitutively expressed genes. Within Antarctic species, high-levels of constitutive expression of CSR genes has been observed in Antarctic ectotherms, a response that is hypothesized to be due to increased levels of oxidative damage [61] and protein damage [62] caused by life at subzero temperatures. In this study, the differential expression of genes within the CSR indicated a mixed physiological response of *L. h. antarctica* to low pH conditions*.*

#### Neural response

In our project on *Limacina*, we specifically explored differential expression patterns in genes involved in neural function because previous work with *H. inflatus* correlated pH exposure to elevated expression of neural genes [37]. In our experiments however, exposure to low pH resulted in the opposite trend - namely, a decrease in expression levels of neural transcripts with the highest percentage found in the low pH treatment on d21 (Fig. 4b). Acute exposure times clearly still grouped the high pH treatment with the T_0_ samples while both the mid and the low pH treatment exhibit a decrease in expression levels (Fig. 4a). Within the up-regulated neural gene set on d21, we found 5 neuronal acetylcholine receptors, and 4 NXPE type-2 transcripts (Additional file 9). These up-regulated transcripts correspond to similar up-regulation of acetylcholine and neural transcripts observed in *H. inflatus*. The down-regulated transcripts included an acetylcholine receptor subunit delta-like, 4 transcripts for small conductance calcium-activated potassium channels and 5 skeletal receptor tyrosine kinase-like transcripts (Additional file 9). The proportion of down-regulated neural transcripts represent a more-pronounced suppression of neural transcripts than was found in the *H. inflatus* transcriptome, potentially due to the shorter exposure time (3 days) and narrower gap in treatment conditions (pH 7.9 vs. pH 8.01). The up-regulation of neuronal receptors coupled with the down-regulation of potassium channels and skeletal tyrosine kinases suggest that exposure to the low-pH treatments will negatively affect locomotion. Here, prolonged exposures to low pH conditions might impact the ability for *L. h. antarctica* to maintain normal neuronal function that could, for example, impede feeding mechanisms and vertical migration under seawater conditions expected in the winter by the year 2050.

### Other studies of comparative transcriptomic and ocean acidification

Other investigators have assessed the impact of *p*CO_2_ levels on the transcriptome of marine invertebrates. These organisms include corals [2], oysters [51], sea urchins [7, 60, 63], mussels [56, 64], and pteropods [22, 23, 37]. In general, our results are in alignment with these other studies, with the general result that changes in *p*CO_2_ exposure does alter the transcriptome. In addition, our results on the Antarctic form of *Limacina helicina* are largely consistent with what has been reported in pteropods as a taxonomic group. Specifically, working with the Arctic form of *Limacina helicina*, Koh and colleagues found significant down-regulation of biomineralization genes [22]. These results on polar *Limacina* spp. contrasts with what has been reported in a distantly related Mediterranean pteropod *H. inflatus* where low pH exposure elicited an up-regulation of biomineralization-related genes [37]. Within the *Limacina* complex this observed divergence in response suggests pteropods from the Southern Ocean may be more sensitive then the temperate pteropod *H. infaltus* to under-saturation conditions of ocean acidification, and did not maintain expression of calcification-related genes when exposed to low pH conditions. Lastly, although the effects of pH on calcification in *Limacina spp.* have been well documented [13, 16, 20, 21, 65–67], there remains an ongoing debate regarding the ability for *Limacina spp.* to utilize internal shell repair mechanisms to minimize the deleterious impacts of ocean acidification [65, 68, 69]. However, this study along with the transcriptome study presented by Koh et al. [22] suggests that all forms of *Limacina helicina* are challenged to maintain calcification in low pH conditions.

## Conclusion

In the present study, we have reported significant changes in the transcriptome of juvenile *Limacina helicina antarctica* in response to *p*CO_2_ conditions in the laboratory that mimics present-day and future conditions. The shell-forming pteropod, *L. h. antarctica*, exhibited sensitivity to low pH stress with significant down-regulation of genes involved in shell formation, metabolism, and the cellular stress response. These results from experiments suggest that contemporary *Limacina* did not increase the expression of genes involved in shell formation in order to compensate for the physiological stress induced by ocean acidification in situ. These results extend our understanding of how this key Antarctic zooplankton species will respond to prolonged exposure to low pH conditions predicted to occur by the winter of 2050. In addition, this study presents an ideal framework from which hypothesis-driven functional validation studies of these effected pathways can be developed.

## Additional files

Additional file 1: Transcriptome assembly and annotation statistics. This file provides further assembly and annotation statistics for the de novo transcriptome. File includes: assembly statistics, gene ontology distribution, and KEGG enzyme mapping. (PDF 117 kb)

Additional file 2: Seasonal differential expression gene list. This file provides the list of all genes identified in the seasonal differential gene expression analysis. File contains, sequence identifier, sequence description, enzyme codes, gene ontology identifiers, gene ontology names, logFC (Fold Change), logCPM (Counts per million), *P*-values, and FDR (false discovery rate). (XLSX 322 kb)

Additional file 3: Ocean acidification differential expression gene list. This file provides the list of all genes identified in the ocean acidification differential gene expression analysis. File contains, sequence identifier, sequence description, enzyme codes, gene ontology identifiers, gene ontology names, logFC (Fold Change), logCPM (Counts per million), P-values, and FDR (false discovery rate). (XLSX 741 kb)

Additional file 4: Seasonal enriched gene ontologies. This file provides the list of all gene ontologies identified as enriched in the seasonal differential gene expression analysis using the Fisher’s Exact Test. File contains: pH comparison, day, gene ontology ID, gene ontology name, FDR (false discovery rate), P-value, Gene ontology category, number of transcripts in test (i.e. those that were differentially expressed), and number of transcripts in the reference transcriptome. (XLSX 11 kb)

Additional file 5: Ocean acidification enriched gene ontologies. This file provides the list of all gene ontologies identified as enriched in the ocean acidification differential gene expression analysis using the Fisher’s Exact Test. File contains: pH comparison, day, gene ontology ID, gene ontology name, FDR (false discovery rate), P-value, Gene ontology category, number of transcripts in test (i.e. those that were differentially expressed), and number of transcripts in the reference transcriptome. (XLSX 56 kb)

Additional file 6: Gene identifiers and calculated z-scores for shell formation analysis. This file provides the list and z-score of all genes identified in the shell formation analysis. File contains, sequence identifier, sequence description, enzyme codes, gene ontology identifiers, gene ontology names, gene ontology category, and z-scores for each sample. (XLSX 366 kb)

Additional file 7: Gene identifiers and calculated z-scores for the cellular stress response analysis. This file provides the list and z-score of all genes identified in the cellular stress response gene analysis. File contains, sequence identifier, sequence description, enzyme codes, gene ontology identifiers, gene ontology names, gene ontology category, and z-scores for each sample. (XLSX 176 kb)

Additional file 8: Gene identifiers and calculated z-scores for the metabolic gene analysis. This file provides the list and z-score of all genes identified in the metabolism analysis. File contains, sequence identifier, sequence description, enzyme codes, gene ontology identifiers, gene ontology names, gene ontology category, and z-scores for each sample. (XLSX 549 kb)

Additional file 9: Gene identifiers and calculated z-scores for the neural gene analysis. This file provides the list and z-score of all genes identified in the neural gene analysis. File contains, sequence identifier, sequence description, enzyme codes, gene ontology identifiers, gene ontology names, gene ontology category, and z-scores for each sample. (XLSX 39 kb)

## Abbreviations

bp: Base pair
CO_2_: Carbon dioxide
CSR: Cellular stress response
FPKM: Fragments per kilobase of transcript per million mapped reads
GO: Gene ontology
HSP: Heat shock proteins
KEGG: Kyoto Encyclopedia of Genes and Genomes
NCBI: NATIONAL Center for Biotechnology Information
OA: Ocean acidification
pCO_2_: Partial pressure of carbon dioxide
RNA-Seq: RNA sequencing
