# Supplementary material for: Transcriptomic response of the Antarctic pteropod Limacina helicina antarctica to ocean acidification
Source: BMC Genomics. 2017 Oct 23;18:812. doi: 10.1186/s12864-017-4161-0 (PMC5653985; doi:10.1186/s12864-017-4161-0)
Supplement: Supplementary file 1 — Transcriptome assembly and annotation statistics. This file provides further assembly and annotation statistics for the de novo transcriptome. File includes: assembly statistics, gene ontology distribution, and KEGG enzyme mapping. (PDF 117 kb) [file 12864_2017_4161_MOESM1_ESM.pdf]

The de novo *Limacina helicina antarctica* transcriptome utilized in these analysis was assembled using Trinity vs. 2.2.0. The final assembly contained 403,210 contigs. To reduce the number of misaligned reads, the analysis was restricted to focusing on contigs that had more then 1 count per million reads in 9 of the 39 samples. This reduced set of "expressed" candidate sequences were utilized in the subsequent annotation and gene expression analysis. This filtering had a significant impact on the transcriptome by reducing the number of assembled contigs to 83,211 sequences. Average contig length increased to 1,281.52 base-pairs, the GC percentage was 41.27%, and the minimum sequence length was 224 base-pairs (Table 1). Blastx query of this reduced transcriptome resulted in the annotation of 37,517 (45.1%) of these transcripts. Gene ontology annotations were added to these annotations resulting in a total of 77,476 gene ontological terms being assigned to 28,407 of the annotated transcripts. The top 20 gene ontologies for the three major categories are presented in table 3. KEGG enzyme mapping assigned 417 enzymes associated with 130 KEGG pathways. The complete list of KEGG pathways is available in table 4.

Table 1. Transcriptome assembly statistics

**Statistic**	**Total**
Contigs	83,211
Total base pairs	216,717,523 bp
Percent GC	41.27%
Contig N50	2,177 bp
Median contig length	1,004 bp
Average contig length	1,425.81 bp
Maximum contig length	19,953 bp
Minimum contig length	224 bp

Table 2. Biological Process Gene Ontologies

Gene Ontology	Number of Sequences
oxidation-reduction process	624
transport	498
translation	418
metabolic process	401
protein phosphorylation	359
regulation of transcription, DNA-templated	353
transmembrane transport	308
phosphorylation	293
single-organism cellular process	283
proteolysis	260
cellular process	259
regulation of cellular process	249
small GTPase mediated signal transduction	242
protein folding	210
microtubule-based process	208
signal transduction	203
single-organism process	190
microtubule-based movement	187
intracellular protein transport	162
carbohydrate metabolic process	153
vesicle-mediated transport	148
intracellular signal transduction	140
protein ubiquitination	137
positive regulation of transcription from RNA polymerase II promoter	125
mRNA splicing, via spliceosome	124
biological regulation	121
protein transport	120
protein dephosphorylation	120
DNA repair	116
methylation	115
primary metabolic process	107
regulation of transcription from RNA polymerase II promoter	102
translational initiation	101
ubiquitin-dependent protein catabolic process	98
ion transport	91

Table 3. Molecular Function Gene Ontologies

Gene Ontology	Number of Sequences
ATP binding	1682
metal ion binding	1018
nucleotide binding	822
RNA binding	660
GTP binding	619
binding	618
zinc ion binding	565
nucleic acid binding	513
protein binding	503
structural constituent of ribosome	443
DNA binding	441
catalytic activity	434
hydrolase activity	411
calcium ion binding	369
GTPase activity	350
transferase activity	329
oxidoreductase activity	314
protein serine/threonine kinase activity	261
kinase activity	235
ATPase activity	218
structural constituent of cytoskeleton	215
organic cyclic compound binding	204
heterocyclic compound binding	202
microtubule motor activity	197
transcription factor activity, sequence-specific DNA binding	182
actin binding	162
sequence-specific DNA binding	157
magnesium ion binding	152
protein kinase activity	151
ligase activity	137
peptidase activity	137
unfolded protein binding	136
ubiquitin protein ligase binding	134
ubiquitin-protein transferase activity	131
flavin adenine dinucleotide binding	129

Table 4. Cellular Component Gene Ontologies

Gene Ontology	Number of Sequences
membrane	1973
integral component of membrane	1876
cytoplasm	1257
nucleus	1241
cytosol	605
intracellular	510
extracellular exosome	445
mitochondrion	421
nucleoplasm	350
intracellular part	331
plasma membrane	312
microtubule	270
nucleolus	223
ribosome	217
intracellular membrane-bounded organelle	176
endoplasmic reticulum	158
cytoplasmic part	157
Golgi apparatus	155
mitochondrial inner membrane	153
cytosolic large ribosomal subunit	137
cell part	132
dynein complex	126
centrosome	115
intracellular ribonucleoprotein complex	110
endoplasmic reticulum membrane	110
cytoskeleton	107
integral component of plasma membrane	107
extracellular space	101
intracellular organelle	98
membrane part	98
extracellular region	95
catalytic step 2 spliceosome	91
myosin complex	86
cell	86
Golgi membrane	84

Table 5. KEGG pathways in transcriptome

Pathway	KEGG Pathway ID	Number of Enzymes in Pathway
Biosynthesis of antibiotics	map01130	110
Purine metabolism	map00230	43
Amino sugar and nucleotide sugar metabolism	map00520	25
Cysteine and methionine metabolism	map00270	25
Pyrimidine metabolism	map00240	23
Glycolysis / Gluconeogenesis	map00010	21
Alanine, aspartate and glutamate metabolism	map00250	21
Aminoacyl-tRNA biosynthesis	map00970	20
Fructose and mannose metabolism	map00051	20
Glycine, serine and threonine metabolism	map00260	20
Valine, leucine and isoleucine degradation	map00280	18
Arginine and proline metabolism	map00330	18
Pyruvate metabolism	map00620	17
Carbon fixation pathways in prokaryotes	map00720	17
Phosphatidylinositol signaling system	map04070	16
Citrate cycle (TCA cycle)	map00020	16
Inositol phosphate metabolism	map00562	15
Pentose phosphate pathway	map00030	14
Glycerophospholipid metabolism	map00564	14
Butanoate metabolism	map00650	13
Carbon fixation in photosynthetic organisms	map00710	13
Propanoate metabolism	map00640	13
Glyoxylate and dicarboxylate metabolism	map00630	12
Tryptophan metabolism	map00380	12
Methane metabolism	map00680	12
Glutathione metabolism	map00480	12
Tyrosine metabolism	map00350	12
Starch and sucrose metabolism	map00500	12
Fatty acid degradation	map00071	11
Drug metabolism - other enzymes	map00983	11
Porphyrin and chlorophyll metabolism	map00860	11
Galactose metabolism	map00052	11
Pentose and glucuronate interconversions	map00040	11
N-Glycan biosynthesis	map00510	10
Lysine degradation	map00310	10
Nicotinate and nicotinamide metabolism	map00760	10
Arginine biosynthesis	map00220	10
One carbon pool by folate	map00670	10

Terpenoid backbone biosynthesis	map00900	10
beta-Alanine metabolism	map00410	9
Fatty acid biosynthesis	map00061	9
Phenylalanine metabolism	map00360	9
Glycerolipid metabolism	map00561	8
Oxidative phosphorylation	map00190	7
Fatty acid elongation	map00062	7
Biosynthesis of unsaturated fatty acids	map01040	7
Sphingolipid metabolism	map00600	7
Pantothenate and CoA biosynthesis	map00770	6
Other glycan degradation	map00511	6
alpha-Linolenic acid metabolism	map00592	6
Histidine metabolism	map00340	6
Various types of N-glycan biosynthesis	map00513	6
Sulfur metabolism	map00920	6
Metabolism of xenobiotics by cytochrome P450	map00980	5
Glycosaminoglycan degradation	map00531	5
Streptomycin biosynthesis	map00521	5
Synthesis and degradation of ketone bodies	map00072	5
Phenylalanine, tyrosine and tryptophan biosynthesis	map00400	5
Drug metabolism - cytochrome P450	map00982	5
Riboflavin metabolism	map00740	5
Ascorbate and aldarate metabolism	map00053	5
Novobiocin biosynthesis	map00401	4
Tropane, piperidine and pyridine alkaloid biosynthesis	map00960	4
Biotin metabolism	map00780	4
Vitamin B6 metabolism	map00750	4
Benzoate degradation	map00362	4
Nitrogen metabolism	map00910	4
Geraniol degradation	map00281	4
Folate biosynthesis	map00790	4
Isoquinoline alkaloid biosynthesis	map00950	4
Arachidonic acid metabolism	map00590	4
Retinol metabolism	map00830	4
Styrene degradation	map00643	3
mTOR signaling pathway	map04150	3
Aminobenzoate degradation	map00627	3
Steroid biosynthesis	map00100	3
Selenocompound metabolism	map00450	3
Caprolactam degradation	map00930	3
Steroid hormone biosynthesis	map00140	3

Ether lipid metabolism	map00565	3
D-Glutamine and D-glutamate metabolism	map00471	3
Thiamine metabolism	map00730	3
Valine, leucine and isoleucine biosynthesis	map00290	2
Phosphonate and phosphinate metabolism	map00440	2
Linoleic acid metabolism	map00591	2
D-Arginine and D-ornithine metabolism	map00472	2
Lysine biosynthesis	map00300	2
Ubiquinone and other terpenoid-quinone biosynthesis	map00130	2
Chloroalkane and chloroalkene degradation	map00625	2
Mucin type O-glycan biosynthesis	map00512	2
Limonene and pinene degradation	map00903	2
Mannose type O-glycan biosynthesis	map00515	2
Cyanoamino acid metabolism	map00460	2
Neomycin, kanamycin and gentamicin biosynthesis	map00524	2
Steroid degradation	map00984	2
Glycosphingolipid biosynthesis - globo and isoglobo series	map00603	2
T cell receptor signaling pathway	map04660	2
Indole alkaloid biosynthesis	map00901	2
Carbapenem biosynthesis	map00332	2
Caffeine metabolism	map00232	1
Monobactam biosynthesis	map00261	1
Glycosphingolipid biosynthesis - ganglio series	map00604	1
Lipoic acid metabolism	map00785	1
Toluene degradation	map00623	1
Other types of O-glycan biosynthesis	map00514	1
Taurine and hypotaurine metabolism	map00430	1
Glycosaminoglycan biosynthesis - keratan sulfate	map00533	1
Flavone and flavonol biosynthesis	map00944	1
Th1 and Th2 cell differentiation	map04658	1
Sesquiterpenoid and triterpenoid biosynthesis	map00909	1
C5-Branched dibasic acid metabolism	map00660	1
Primary bile acid biosynthesis	map00120	1
Insect hormone biosynthesis	map00981	1
PI3K-Akt signaling pathway	map04151	1
Glycosaminoglycan biosynthesis - heparan sulfate / heparin	map00534	1
Betalain biosynthesis	map00965	1
Glycosylphosphatidylinositol (GPI)-anchor biosynthesis	map00563	1
Penicillin and cephalosporin biosynthesis	map00311	1
Human papillomavirus infection	map05165	1
Ethylbenzene degradation	map00642	1

Aflatoxin biosynthesis	map00254	1
Phenylpropanoid biosynthesis	map00940	1
Naphthalene degradation	map00626	1
Glycosaminoglycan biosynthesis - chondroitin sulfate / dermatan sulfate	map00532	1
Diterpenoid biosynthesis	map00904	1
Zeatin biosynthesis	map00908	1
Glucosinolate biosynthesis	map00966	1
Cutin, suberine and wax biosynthesis	map00073	1
Biosynthesis of ansamycins	map01051	1
